# Resistance Training with Blood Flow Restriction and Ocular Health: A Brief Review

**DOI:** 10.3390/jcm11164881

**Published:** 2022-08-19

**Authors:** Michał Krzysztofik, Dorota Zygadło, Paulina Trybek, Jakub Jarosz, Adam Zając, Nicholas Rolnick, Michał Wilk

**Affiliations:** 1 Institute of Sport Sciences, The Jerzy Kukuczka Academy of Physical Education in Katowice, 40-065 Katowice, Poland; 2 Faculty of Science and Technology, University of Silesia in Katowice, 41-500 Chorzów, Poland; 3 The Human Performance Mechanic, CUNY Lehman College, Bronx, New York, NY 10468, USA

**Keywords:** glaucoma, ischemia, occlusion, intraocular pressure, strength training

## Abstract

Despite the many health benefits of resistance training, it has been suggested that high-intensity resistance exercise is associated with acute increases in intraocular pressure which is a significant risk factor for the development of glaucomatous optic nerve damage. Therefore, resistance training using a variety of forms (e.g., resistance bands, free weights, weight machines, and bodyweight) may be harmful to patients with or at risk of glaucoma. An appropriate solution for such people may involve the combination of resistance training and blood flow restriction (BFR). During the last decade, the BFR (a.k.a. occlusion or KAATSU training) method has drawn great interest among health and sports professionals because of the possibility for individuals to improve various areas of fitness and performance at lower exercise intensities. In comparison to studies evaluating the efficiency of BFR in terms of physical performance and body composition changes, there is still a paucity of empirical studies concerning safety, especially regarding ocular health. Although the use of BFR during resistance training seems feasible for glaucoma patients or those at risk of glaucoma, some issues must be investigated and resolved. Therefore, this review provides an overview of the available scientific data describing the influence of resistance training combined with BFR on ocular physiology and points to further directions of research.

## 1. Introduction

Glaucoma poses a serious and increasing problem for public health as it is the leading cause of irreversible blindness and significant reductions in quality of life [1]. Glaucoma is a group of progressive optic neuropathies characterized by a degeneration of retinal ganglion cells and retinal nerve fiber layers that result in changes in the optical nerve head, leading to visual field loss and eventual blindness [2]. Risk factors include age and frailty, gender, myopia, genetics, family history, smoking, race, systemic hypotension and hypertension, vasospasm, use of systemic or topical steroids, migraine, obstructive sleep apnea syndrome, and most significantly, increased intraocular pressure (IOP) [3]. Although the primary strategy for managing glaucoma is based on pharmacological or surgical interventions, other factors such as diet, exercise, or sleeping position should also be considered to reduce the progression of this disease and avoid symptoms (e.g., visual field loss) [2]. Therefore, physical activity needs to be carefully and individually prescribed to induce positive instead of adverse effects on ocular physiology depending on the type of exercise and participants’ characteristics.

A primary exercise intervention for muscle and strength development is resistance training [4]. Regular resistance training provides several benefits for the human body such as improving body composition, glycemic control, cardiovascular health, increasing bone mineral density, facilitating physical functions, and enhancing mental health [5]. Therefore, this type of physical activity is readily practiced by populations of all ages and is recommended as a part of the World Health Organization 2020 guidelines [6]. This also applies to the elderly, who can effectively counteract or reverse the aging process with resistance training [7]. This type of training can be accomplished using bodyweight, resistance bands, free weights, or weight machines. Resistance bands and bodyweight training may provide an alternative when traditional weight equipment is unavailable. However, determining the appropriate training intensity and maintaining progression over time, as well as achieving high involvement of particular lower body muscles are more challenging with these forms [8]. Therefore, free weight and weight machine training should be prioritized and for optimal benefits, the American College of Sports Medicine (ACSM) recommends 1−3 sets per exercise of 8−12 repetitions with 70−85% of one repetition maximum (1RM) for novice and 3−6 sets of 1−12 repetitions with 70−100% 1RM for advanced trainees [9]. However, for many populations, the above-mentioned training demands while recovering from injuries or undergoing rehabilitation and dealing with different forms of chronic inflammation and pain (e.g., arthritis) may make adherence to this recommendation challenging. Moreover, it may be potentially harmful to glaucoma patients, especially given that people over 60 years of age are at an increased risk of glaucoma and have to counteract the progressive loss of muscle mass and strength with age [10].

Even though resistance training offers a variety of positive health effects, it has been hypothesized that high-intensity resistance training may cause acute increases in IOP [11,12,13,14], which is a substantial risk factor for the onset of glaucomatous optic nerve injury [15]. Therefore, resistance training following ACSM guidelines may be harmful to patients with or at risk of glaucoma. According to the available data, increases in IOP after high-intensity resistance training vary on the exercise and load applied, with heavier loads and exercises involving several muscle groups showing the greatest alterations [14,16,17]. This phenomenon may be related to core stability or bracing during high-intensity exercise that is achieved by trunk muscle contraction and often results in a Valsalva maneuver [18]. Consequently, the increases in intra-abdominal and intrathoracic pressures are transmitted to systemic vascular and intracranial transmural pressures [19], affecting IOP. In addition, body positioning during exercise also influences IOP levels, with greater IOP values in supine than sitting or standing [20]. This creates significant limitations in terms of designing and progressing resistance training programs, especially in at-risk patients beginning a resistance training program. However, it is also worth noting that the IOP response is reduced in trained individuals, indicating that fitness levels may modulate this phenomenon [13]. Hence, this may be related to several adaptive processes which accompany regular resistance training, highlighting the importance of considering training status to prevent/reduce undesirable effects on ocular health and gradual progression models recommended by the ACSM.

Considering the above, an appropriate solution for glaucoma patients or those at risk of glaucoma may involve the combination of resistance training and blood flow restriction (BFR). This training solution involves the use of an inflatable cuff, tourniquet, or elastic wraps that exert high pressure at the proximal part of the limb (lower or upper), to reduce arterial blood flow and to occlude venous blood flow during physical exercise. Resistance training loads with BFR exercise are reduced due to increased metabolic stress [21], cell swelling [22], intramuscular signaling [23], and enhanced endocrine system responses from the reduction of arterial inflow to the exercising limb [24]. Hence, while maintaining similar effectiveness to high-intensity resistance training in terms of muscle mass and strength [25], an adjunctive benefit of BFR exercise may be a potential reduction of IOP secondary to reduced training loads (20–30% 1RM). Therefore, this review provides an overview of the available scientific data describing the influence of resistance training combined with BFR on ocular physiology and points to further directions of research.

## 2. Literature Search

PubMed and Scopus databases were searched for all studies investigating blood flow restriction and ocular health. The search was performed using the following keyword combinations: (“blood flow restriction” OR “occlusion”) AND (“safety” OR “ocular health” OR “glaucoma” OR “intraocular pressure”). The present review includes studies that (i) presented original research data on healthy adult subjects, (ii) were published in peer-reviewed journals, and (iii) were published in the English language. There were no gender and age restrictions imposed.

## 3. Safety Concerns and Blood Flow Restriction Resistance Training

The BFR method has been used to an increased extent for different purposes. Commonly cited approaches include increasing rehabilitation potential [26], improving athletic performance [27,28], and improving general health [29]. Therefore, different groups of individuals and a wide array of BFR protocols have been examined [30,31,32,33]. However, most of these studies have enrolled healthy participants with a focus on physical performance outcomes and body composition change [23,34,35] or physiological responses [24,36,37]. On the other hand, only a few studies have focused on assessing the adverse effects of long-term BFR training [33,38,39,40]. Therefore, given the growing interest in the BFR method in various human populations, verification and documentation of safety become urgent.

The phrase “blood flow restriction” includes nearly 11,000 articles in PubMed, and adding “safety” reduces that number to 254. A significant part deals with theoretical considerations, literature reviews, and meta-analyses; excluding those studies from the search, only 34 original articles devoted to the BFR resistance training safety remain. Therefore, in comparison to studies evaluating the efficiency of BFR in terms of physical performance and body composition changes, there is still a paucity of empirical studies concerning safety, especially regarding ocular health. Nonetheless, studies to date report negligible adverse effects due to the use of BFR [41,42,43]. Probably the largest survey was conducted in 2005 [41] and repeated in 2016 [42] in Japan. In each, responses from over 12,000 participants from 105 and 232 facilities were collected, respectively. The first study found an incidental occurrence of serious side effects as follows: venous thrombus (0.055%), pulmonary embolism (0.008%), and rhabdomyolysis (0.008%). However, in the latter, such side effects were not noticed. This may mean that knowledge about BFR training is gradually increasing, reducing the risk of severe side effects. In 2011, Leonneke and colleagues [44] summarized the safety of BFR training, pointing out that possible concerns may be related to training-induced alternations in cardiovascular functions and the peripheral nervous system. Attention is also paid to the potential increased oxidative stress or the response of antioxidant enzymes [45], and muscle damage [43,46]. Considering ocular function, BFR resistance training may indirectly impact changes in systemic vascular pressure. Hypoxia and accumulation of metabolites in active muscle during BFR resistance training might augment the metaboreflex resulting in increased blood pressure. This is supported by evidence showing that regular BFR resistance training significantly increases blood pressure compared to traditional resistance training [47,48,49]. In addition, this phenomenon might be pronounced in hypertensive individuals [48]. However, it is possible that the blood pressure changes could be pressure-dependent. The pilot study by Maciel et al. [50] reported an acute reduction of blood pressure after BFR resistance training at 60% of arterial occlusion pressure (AOP) but increased systolic blood pressure by 15 mmHg after BFR with 80% of AOP. Nonetheless, high and low blood pressure is associated with an increased risk of glaucoma [51].

Although using BFR during resistance training seems feasible for glaucoma patients or those at risk of glaucoma, some issues must be investigated and resolved. First, although the effects of resistance training on ocular health have been studied [11,12,13,52], there is only one pre-preliminary study using BFR [14]. In addition, this study examined BFR without resistance exercise and with a short restriction duration (only 40 s) using both high (60% AOP) and low pressures (40% AOP) in the upper and lower limbs [14]. BFR without exercise with such short restriction times is not an often-used approach, and recent recommendations indicate that the duration of the restriction should be between 5–10 min per exercise [29]. Nevertheless, a study by Vera et al. [14] provides interesting results that are worth discussing from the perspective of future research. Authors found that IOP and ocular perfusion pressure did not change significantly during BFR applied on lower limbs with both high and low pressure as well as during upper limbs with low pressure (no difference between sexes). However, high-pressure upper limbs BFR induced a significant increase (for both sexes) in IOP and decreased ocular perfusion pressure (only for men). Moreover, only relatively lower AOPs (a measure used to prescribe intensity of restriction) during BFR were examined, while a greater level of 80% falls within the current recommendations [29]. Overall, it seems that an increase in IOP due to BFR might depend on the applied pressure and place of application, while decreases in ocular perfusion pressure could be sex-dependent. Most importantly, the long-term effects of BFR on vision health are unknown.

## 4. Potential Benefits of Blood Flow Restriction Resistance Training

The physiological mechanisms underlying muscle adaptation from BFR are widely studied, and it seems that increased fast-twitch fiber recruitment [53,54,55], decreased myostatin expression [56], satellite cell proliferation [57], and acute muscle cell swelling may contribute to the anabolic response of BFR during low-load resistance training [22]. Recent literature has shown that low-load BFR resistance training outperforms body-weight or low-load training without BFR [34]. Moreover, it may even produce effects similar to moderate-heavy resistance training, including increases in muscle strength and muscle mass [34], cardiovascular capacity [58], and performance in specific tasks [59]. Therefore, if BFR resistance training would be deemed a safe alternative to high-intensity strength training for glaucoma patients and those at risk for glaucoma, it could mitigate the potential side effects of high-intensity resistance training while still reaping its benefits. Furthermore, it might also make multi-joint exercises less demanding. This may be significant since multi-joint exercises involving large muscle groups seem superior in improving general fitness [60] but induce higher IOP increments and ocular perfusion pressure reduction than single-joint exercises [16]. In addition, during high-intensity resistance exercise, it may be difficult to control the correct breathing technique and, consequently, increase IOP due to holding the breath or using the Valsalva maneuver [61]. However, to the authors’ knowledge, no study has assessed the long-term effects of BFR on eye physiology, and only one study has assessed the immediate effect [14]. A study by Vera et al. [14] (described in detail above) highlights a gap in the literature and gives new perspectives for further studies on the impact of BFR on ocular health. Given the strong evidence that BFR resistance training might have unique benefits for the elderly yet considering that eye diseases such as glaucoma risk increases with age, there is a need to understand the impact of BFR on eye health.

## 5. Prospects for Future Insights

Due to the lack of evidence, it is not possible to conclude whether patients with glaucoma or those at risk of glaucoma can safely benefit from using BFR. Therefore, a comprehensive examination of this method and confirmation of beliefs is critical, especially since it is being used by an increasing number of populations and may play an essential role in managing glaucoma. However, based on the outcomes of studies on BFR and ocular health, several points can be raised concerning future experimental designs. Restriction time similar to the recommended durations (e.g., no more than 10 min during resistance exercise) with a wider range of total AOPs (from 40% to 80%) would help establish whether BFR alone might be feasible in terms of glaucoma management. Another issue that will need to be addressed is the impact of low-load BFR resistance training on IOP and ocular perfusion pressure, considering the variables mentioned above of BFR together with other training variables (i.e., training volume, exercise selection, movement tempo, rest intervals). This is particularly relevant given that some studies show exercise type (multi- vs. single-joint) dependent responses when performed until failure [16,17] during traditional high-intensity resistance training.

Moreover, the influence of body position on IOP [2] should be considered as it may not exhibit similar changes as AOP (e.g., becoming larger when transitioning from supine to sitting to standing, respectively). For instance, Najmanova et al. [62] showed significantly higher IOP in the lying compared with the sitting position, diverging from what would be observed with AOP. Therefore, when selecting exercises, trainers and investigators should pay attention to whether they are performed in a lying or sitting position, and it seems that exercises with the head down should be avoided. Further, correct breathing techniques (e.g., not holding breaths in during training) may be an important factor to minimize increases in IOP. Increasing breathing control with simultaneously reduced breath holding (as commonly observed in traditional resistance training) can be obtained through the use of a controlled, slow movement tempo during resistance exercise where inhalation and exhalation phases are performed independently of the contraction of the movement [63,64].

Besides this, different modes of BFR application should be examined [29]. There are four different methods of BFR training as part of physical exercise: pre-condition (ischemia used only before training session), continuous (ischemia used during effort and rest periods), intermittent (used only during effort), and intra-conditioning/resting BFR (used only during rest periods). Although intermittent BFR can be cumbersome due to the need to constantly release and inflate the cuff, it could provide a meaningful alternative to continuous application with less risk of possible adverse effects [33], thus further in-depth research into these areas is urgently needed. Currently, there are no guidelines and safety rules for BFR training using various application methods. Referring to the same safety guidelines when using intermittent and continuous BFR training methods may not produce equivocal acute physiologic stress and may be tolerated differently in populations with ocular problems. The relationship between the type of training method, the BFR pressure used, the type of exercise used, and the starting position (e.g., lying vs. standing) when glaucoma patients perform exercise cannot be ignored in programming safe BFR training. It can be assumed that for glaucoma patients as well as for other people with different dysfunctions, the use of intermittent BFR applied during the rest periods only will involve a lower risk when cuffs are applied during exercise. Of course, the chronic effects following intermittent BFR training may also be different and should be investigated [65]. However, there are no studies that compare the chronic effects of different BFR training methods on muscle adaptation, and what is more, there are no formal recommended safety guidelines for clinical integration for populations with comorbidities including glaucoma. The evidence thus far has shown similar gains in muscle strength, endurance, hypertrophy, and physiological responses [33,66] between application approaches. However, in at-risk populations including those with ocular problems, continuous BFR application may unnecessarily heighten safety risk, necessitating other approaches.

Another important BFR application parameter that should be considered is the influence of different restrictive cuff widths on alterations in vision health. This is important since different cuffs are used for BFR and narrow cuffs (e.g., 5 cm) require higher pressure to occlude blood flow than wider ones (e.g., 13.5 cm) [67]. Moreover, Rossow et al. [68] revealed greater blood pressure responses applying wider cuffs compared with narrower ones; however, the same absolute degree of restriction (152 vs. 154 mmHg) was used for both conditions but was not standardized to personalized AOP. Although when restriction is made relative to AOP, cuff width might not significantly affect physiological responses, some points should be addressed for further clarification. On the one hand, using a wide cuff increases the distance of pressure being applied to the tissue and blood vessels, creating greater resistance to blood flow [69]. Conversely, a narrow cuff might increase the risk of nerve injuries due to a higher-pressure gradient (since high AOP is required) compared with wider cuffs [69,70]. Therefore, to clarify this issue, further studies should compare the impact of wide and narrow cuffs with equal percent of AOP on IOP and ocular perfusion pressure. Finally, once the safest settings have been established, long-term investigations with outcomes monitoring [71,72] should be introduced to verify and document the safety of BFR use.

## 6. Conclusions

The evidence provided in the present article questions the ocular safety of glaucoma patients when BFR is combined with low-load resistance training due to a limited number of studies, requiring further investigations. This information is paramount for the consideration of BFR resistance training implementation in glaucoma populations. Accordingly, we recommend that investigators, therapists, and trainers carefully introduce BFR resistance training protocols in patients with or at risk of glaucoma or wait until more empirical evidence becomes available before integrating them into practice. Therefore, if someone decides to use BFR, clinicians should consider settings that could potentially be the least harmful such as low-load single-joint exercises with lesser applied BFR pressures in sitting or standing positions, and draw attention to early signs of complications.

## Data Availability

Not applicable.
